# Climate adaptation in the public health sector in Africa: Evidence from United Nations Framework Convention on Climate Change National Communications

**DOI:** 10.4102/jamba.v11i1.644

**Published:** 2019-04-09

**Authors:** Godwell Nhamo, Shepherd Muchuru

**Affiliations:** 1 Institute for Corporate Citizenship, University of South Africa, Pretoria, South Africa

**Keywords:** Africa, adaptation, climate change, health, sustainable, UNFCCC

## Abstract

Climate change has potential to affect human health in various ways. Extreme temperatures and cold both result in deaths, while the changing habitats favouring the breeding of vectors could result in the spread of diseases such as malaria, cholera and typhus. This article reviews climate change adaptation measures in the African public health sector. The evidence is drawn from National Communications of 21 countries as submitted to the United Nations Framework Convention on Climate Change (UNFCCC). This article combines the literature review and grounded theory approaches with data obtained from the UNFCCC National Communications. Among key adaptation measures emerging from the work are weather-based forecasting and early warning systems, public education and awareness, putting in place appropriate policies, surveillance, research and monitoring as well as improving public health infrastructure and technology. The study recommends that African nations should commit to address health impacts of climate change through the implementation of appropriate adaptation measures.

## Introduction

Climate change is real, evident and convincing. Significant research has been conducted on climate change and its impact on human health, revealing the vulnerability of developing nations to the phenomenon (Haines et al. 2009). The changing climate is evident by rising sea level, increasing temperatures, hydrological cycle modification and increased climate variability (droughts, hailstorms, floods and extreme heat, cold and veld fires). Many such extreme weather events significantly impact human health. Among the key health risks identifiable are air pollution, increased susceptibility to infectious and animal vectors causing diseases, food and water stress as well as displacement of population. Such health risks cause malnutrition, with more than 18% of the affected population in developing nations (Food and Agricultural Organisation [FAO] 2005). The reasons for such health risks include weak public health systems (Ford 2012) coupled with high poverty levels and inequality, low technology use and innovation and weak institutions that exacerbate vulnerability (Walpole, Rasanathan & Campbell-Lendrum 2009). To this end, if climate change impacts are not dealt with intensively, they could hinder the attainment of the sustainable development goals (SDGs) in general (Nhamo 2017) and those SDGs related to health.

A question then arises as follows: what adaptation measures exist in the public health sector in response to the changing climate in African countries as portrayed in the United Nations Framework Convention on Climate Change (UNFCCC) National Communications? This article reviews climate change adaptation measures emerging from the UNFCCC National Communications from 21 African countries. Such adaptation measures will benefit the present and future generations if implemented in spite of severity of the health impacts of climate change.

### Literature review

According to Haines et al. (2006), climate change is reportedly claiming over 150 000 lives and 5.5 million disability-adjusted life years per year since 1970 globally. The Intergovernmental Panel on Climate Change (IPCC) has estimated further deterioration of the health sector in the near future. The Fifth Assessment Report of the IPCC-AR5 concluded that, globally, rising temperatures and changing rainfall patterns were linked to a range of health outcomes (IPCC 2014). Climate change-related alterations in weather and climate patterns would affect many determinants of health like thermal stress, floods, storms, droughts, bushfires, and food and water quality and quantity. Climate change will further affect the geographic range and activity of infectious diseases. The climate change impacts are associated with the time period between 2030 and 2050 (Smith et al. 2014). In the assessment, sub-Saharan Africa (SSA) is projected to have the greatest burden of mortality impacts attributable to climate change by 2030 (World Health Organization [WHO] 2009). Furthermore, WHO estimates 38 000 deaths are projected for the year 2030 as a result of extreme heat in elderly people, 60 000 because of malaria, 48 000 because of diarrhoea and 95 000 because of childhood undernutrition (WHO 2009). The projections show a dramatic increase in climate and extreme weather events-related deaths by the 2050s, with heat exposure resulting in over 100 000 deaths annually (WHO 2009).

Climate change is prompted to exacerbate infectious, food and vector-borne diseases, and other communicable diseases (Campbell-Lendrum et al. 2015). From Papworth, Maslim & Randalls (2015) observations, climate change will negatively impact access to health services and nutritional security. Such health impacts may be direct, through the outbreak of diseases among the human population, or can be indirect, in terms of outbreaks that impact plants and domesticated animals (Springmann et al. 2016). These negative impacts on the health system will likely affect trade, agriculture and ultimately food security. This has raised concern to focus on the spread and future distribution of infectious diseases with population increase and mobility (Tatem 2014). The changing climate is also known for causing environmental pollution that threatens human health. Accumulation of harmful chemicals in human bodies through air, food and water may lead to diseases such as cardiovascular diseases, cancers and diabetes (Depledge et al. 2013). Other adverse impacts include threat to food availability and water security, all with repercussions on human health.

Africa is more vulnerable to climate change because of increased exposure to environmental threats resulting from the changes in global environment, increasing population growth and low adaptive capacity (Hajat et al. 2014). This leads to two assumptions: firstly, the wide range of health impacts on human health and, secondly, the need to develop appropriate adaptation strategies in the face of the changing climate. Impacts of the changing climate on human health are either direct or indirect. Among the direct impacts are extreme weather events such as cold spells, heat waves, cyclones, droughts and hailstorms (Louis & Hess 2008). Indirect impacts include air pollution, infectious and vector-borne diseases, and all these affect human health (Haines et al. 2006). Adaptation can be defined as the ‘ability of a system (policies, projects and programs) to reduce vulnerability to impacts of climate change’ (Papadaskalopoulou et al. 2015:96). Adaptive capacity in this context refers to the ability of countries to engage in adaptation. To reduce the negative effects of climate change, appropriate and sound adaptation strategies must be employed. The adaption options can be drawn on a range of possible technological, behavioural changes and socio-economic status and among these are improved surveillance of infectious epidemic diseases in potential areas, early warning systems for human health, health vulnerability assessments to climate change, public health and environmental education and institutional coordination and disaster preparedness.

Thus, to make the health sector more resilient to the impacts of climate change, it is imperative to promote enabling environments through well-informed policies in form of adaptation options to prevent health-related diseases and, thus, promoting a sustainable health sector. The next section focuses on the materials and methods used in generating data and analysing the findings.

## Research methods and design

This article answers the following question: what adaptation measures exist in the public health sector in response to the changing climate in African countries? The question was answered through reviewing adaptation measures from 21 African countries based on their National Communications to the UNFCCC with the objective to outline national progress on implementing the convention. A systematic approach was employed through coding using both targeted health vulnerabilities and typology of adaptive measures.

The 21 countries purposefully sampled include Botswana, Egypt, Eritrea, Gambia, Ghana, Guinea-Bissau, Kenya, Lesotho, Malawi, Mauritius, Namibia, Nigeria, Rwanda, Sierra Leone, South Africa, Sudan, Swaziland, Seychelles, Uganda, Zambia and Zimbabwe. The countries were sampled based on certain criteria. Firstly, those with the most updated National Communications in the form of either the second or third National Communication report submitted to the UNFCCC (see Table 1). The reason for considering the second or third National communications was to gather the latest available data from the selected countries. Secondly, the National Communications selected were those from English-speaking countries as the authors can only read and understand English.

**TABLE 1:** Sampled countries and National Communications chosen.

Country	Second National Communication	Third National Communication
Botswana	2011	-
Egypt	-	2016
Eritrea	2012	-
Gambia	2012	-
Ghana	-	2015
Guinea-Bissau	2011	-
Kenya	2015	-
Lesotho	2014	-
Malawi	2011	-
Mauritius	-	2016
Namibia	2011	-
Nigeria	2014	-
Rwanda	2012	-
Sierra Leone	-	2016
South Africa	2011	-
Sudan	2013	-
Swaziland	2012	-
Seychelles	2011	-
Uganda	2014	-
Zambia	2014	-
Zimbabwe	-	2016

The National Communications reports were retrieved from the UNFCCC website. From the preliminary analysis, 35 categories of adaptive measures in the public health sector emerged and these were later re-organised into six thematic areas, which included early warning systems, public education and awareness, surveillance, research and monitoring, enhanced infectious disease control programmes, policies development, improved public health infrastructure and technology. Details of the themes and number of categories in each of the themes are presented in Table 2.

**TABLE 2:** Emerging themes and categories of the reviewed adaptation measures.

Theme	Categories
Early warning systems	The development of weather and seasonal forecasting and early warning systems Developing control programmes for infectious diseases Enhancing early warning on weather changes Strengthening hydro-agro-meteorological information warning system Developing risk maps that would allow mapping the areas most exposed to bad weather Introducing health alert networks
Public education and awareness	Provision of public education about the risks and appropriate protective behaviours Promote health education Improving garbage, waste collection and disposal Institutionalising health educational system, training and sensitisation Awareness creation about preventive options Training programmes for health personnel to deal with emerging diseases Promoting appropriate climate-health education in schools
Surveillance, research and monitoring	Strengthen the existing disease surveillance system Promote surveillance and research for disease and vectors Integrated disease surveillance and response Research and modelling Develop and implement more effective surveillance and emergency response systems Developing risk indicators (e.g. mosquito numbers and aeroallergen concentration) health outcomes (e.g. infectious disease outbreaks)
Infectious disease control	Vector control, case detection and treatment Promoting access to quality health services Improving vaccination programmes Improve access to safe/potable water and sanitation Integrated management of childhood infections Provision of malaria control programmes
Policies development	Developing action plans and strategies to control infectious diseases and vectors Developing policy and regulatory framework for disease control Mainstreaming climate change in health policies and strategies
Public health infrastructure and technology	Provision of improved housing insulation including subsidies for lower-income families Promoting engineering interventions such as clean water technologies and bio-latrines Developing improved public health infrastructure Increasing geographical accessibility to health services Encouraging planned housing in urban areas that avoid floodplain areas and swamps Adapting architectural designs to suit new weather regimes Continuous maintenance of national public health infrastructures

Following Patrick and Munro’s (2004) framework, this article also employed a literature survey approach on the impacts of climate change on public health system and a review of online submitted adaptation measures. In addition, the grounded theory approach by Glaser and Strauss (1967), which was documented later by Hussein et al. (2014), was also employed. Grounded theory entails the use of literature as data and in this case, data were retrieved from the National Communications reports. The grounded theory approach aims to find a theory from data systematically obtained from social research. It further explores integral social relationships and provides contextual meaning (Crooks 2001). The meaning was then derived from the retrieved information.

Grounded theory approach provides comparative advantages such as its richness in imaginative concepts that are easy to apply, ability to develop new ideas, and quick and easy for data analysis. Glaser (1978) permits systematic data collection and analysis (Hussein et al. 2014). The grounded theory was derived inductively through systematic collection and analysis of data (Strauss & Corbin 1990). Data were collected and contextualised to give meaning and draw conclusions from the submitted National Communications reports. The adaptation themes and categories of the analysis all come from the National Communications reports. The emerged themes captured the essence of measures and experience drawn from varied country’s submissions and contexts instituted to make the health sector resilient and sustainable in as far as climate change is concerned.

Systemic coding enabled the listing of adaptation categories and reformulating them as they emerge from the National Communications reports. In the National Communications, countries report adaptation priorities within and across many sectors, with health being one of them. All coded actions and data were compared with the aim to enhance validation and accuracy. All statements explaining adaptation options that pointed human health vulnerability and climate change were accepted in a way of facilitating authors’ position in establishing what qualifies as adaptation. The relationship of the emerged categories was analysed through conceptualisation to give groups codes those with similar content (where new concepts are core parameters of the data and codes can be seen as dimensions of these concepts). Policies, projects or programmes containing multiple types of adaptation qualified as categories derived from adaptation themes. The theory was generated through categorising to compare clusters of relationships within the context of the emerging adaptation categories. Theorising constructs system of explanations for the main concerns of the subject of the research (Glaser & Strauss 1967).

The study comprehended some of the limitations that come with employing self-reporting as source material. The methodology, the analysis of self-reports by countries in the UNFCCC, may result in both underinclusive and overinclusive findings (Gosling et al. 2004). Some countries may be more skilful at taking (or motivated to take) credit for measures already taken and characterising them as adaptation, while other countries may be less skilful or motivated at articulating that connection. As such, the study took this into consideration during the design stages. However, as noted by Robins, Norem and Cheek (1999), self-reporting comes with advantages of information richness and its extraordinary practicality. It also presents both an efficient and inexpensive data source (Trapnell 2006) and can be administered in mass testing sessions (as opposed to one-on-one interviews). Hundreds of variables can easily be collected in one sitting.

## Results

There are 51 African countries, which are signatories to the UNFCCC. Of the 51 countries, the study selected 21 English-speaking countries and with the most updated National Communications. Following a careful review of the National Communications, it further emerged that 18 out of the 21 countries were either implementing or considering to implement the adaptation measures from the thematic areas identified (see Table 3). The remaining countries were silent.

**TABLE 3:** Summary status regarding the implementation of adaptation measures.

Country	Categories					
	Early warning systems	Public education and awareness	Surveillance, research and monitoring	Infectious disease control	Policies development	Public health infrastructure and technology
Gambia	-	++	++	+	+	++
Lesotho	++	-	+	-	++	-
Malawi	-	++	-	-	+	-
Mauritius	-	+	+	-	+	-
Nigeria	++	++	-	-	+	-
Rwanda	++	++	-	++	+	-
South Africa	++	-	++	++	++	++
Seychelles	-	-	+	-	+	-
Uganda	+	-	-	++	++	-
Zambia	+	-	-	-	+	-
Egypt	++	-	++	++	++	++
Guinea-Bissau	-	-	-	-	+	-
Eritrea	+	+	++	++	+	+
Sierra Leone	+	+	+	++	+	+
Namibia	-	-	-	-	++	-
Botswana	-	+	++	++	+	-
Zimbabwe	-	-	-	-	+	-
Ghana	-	+	++	-	++	-

++, implemented; +, in consideration.

### Weather-based forecasting and early warning systems

The sampled countries are carrying out intervention measures to make the health sector more sustainable and resilient to the impacts of climate change. A good example is the adaptation showcased by Egypt. The Government of Egypt is developing early warning systems through carrying out seasonal and weather forecasting for disaster planning, well-informed educational and public awareness programmes (Government of Egypt 2016). Follow-up interventions are made by social and health actors to the vulnerable people in a way of achieving appropriate and total responsiveness to the issued warnings. Moreover, the government is developing infectious diseases control programmes informed by the early warning systems. The control programmes address diseases such as lymphatic filariasis, rheumatic heart disease, Rift Valley fever, rheumatic fever, schistosomiasis, tuberculosis, malaria, and water- and food-borne diseases (Government of Egypt 2016).

Similarly, Nigeria is enhancing its seasonal weather forecasting systems by providing up-to-date and relevant information well in advance, such as dry spells, heavy storms and heat waves (Government of Nigeria 2014). The system can issue health alerts through analysing and integrating weather and health thresholds data. The idea is streamlined to deal with various health challenges faced in the country’s ecological zones as a result of the changing climate. The government is also working towards making data available that can be used for analysis (statistical) of climate data. Research is being carried out on infectious diseases, heat wave-related sickness, water- and food-borne diseases, socio-economic effects from extreme weather events including understanding urban vulnerabilities to heat wave and cold spells (Government of Nigeria 2014).

Lesotho is considering developing early warning systems, focusing largely on adverse health-related conditions; enhancing knowledge generation, management and information sharing to improve communication networks. The early warning systems are meant to capacitate the Ministry of Health and Social Welfare (MOHSW) (Government of Lesotho 2014). Rwanda, through its Ministry of Natural Resources, is implementing and strengthening hydro-agro-meteorological early warning system by creating and rehabilitating new hydro-meteorological stations. The country is developing risk maps to enable mapping areas that are prone to extreme weather events, so as to provide alerts to people in advance of imminent disasters like floods and hailstorms. The ministry is also rolling out awareness and capacity building programmes to the public to subscribe to health insurance systems (Government of Rwanda 2012). Similarly, the South African government developed early warning systems to identify possible outbreaks of water-borne diseases. Early warning systems are further used for water monitoring and early corrective action application especially in dealing with freshwater algal blooms (WHO/UNICEF 2010).

Uganda developed its Health Sector Strategic and Investment Plans (HSSP I and HSSP II). One of the key priorities within the strategies is to develop early warning systems that will lead to the dissemination of weather forecasts to communities for timely response and preparedness (Government of Uganda 2014). Zambia, Eritrea and Sierra Leone were considering developing and testing early warning systems coordinated by one national agency and with the involvement of vulnerable communities. Through early warning systems, the governments will be able to promote community early warning on weather changes and to respond in advance for malaria and other diseases outbreaks (Government of Eritrea 2012; Government of Sierra Leone 2016; Government of Zambia 2014).

### Public education, training and awareness

Malawi, through the Ministry of Health and Population Services (MoHPS), is rolling various programmes for educating the public on risks and behavioural change to deal with climate change impacts on its health system. For instance, the government is promoting the use of cooling fans during very hot days, opening windows to allow ventilation, putting on loose-fitting and light clothes, avoiding exertion and if need be water sprinkling on clothes (Government of Malawi 2011). The public is being educated on waste and garbage collection and refusal disposal to avoid breeding of mosquitoes (Government of Malawi 2011). The educational system is being institutionalised with training and sensitisation programmes on health issues. Community Health Workers (CHWs) are deployed in various areas of the country to assist communities on health education. The CHWs are using participatory hygiene and sanitation approaches and hygiene and sanitation in school environment in promoting climate-health education either in communities or in schools (Government of Malawi 2011). A typical example is in Rwanda, where participatory hygiene and sanitation in schools in 2008, reached approximately 78% of the population (Government of Rwanda 2012). Community health workers are capacitated to promote best health educational services to the public. Some of health educational practices being promoted include the use of improved latrines and promote a culture of handwashing after visiting the toilet both in institutions and households (Government of Malawi 2011).

Gambia is continuously providing public health awareness and education programmes. The health awareness programmes are carried out to assist communities to make informed decision on diseases prevention measures. The health promotion and educational programmes are incorporating climate science and addressing the impacts of climate change on both non-infectious and infectious diseases (Government of Gambia 2012). Likewise, Mauritius was considering to conduct regular awareness training programmes for the health officials. Public health awareness programmes are accompanied by simulation exercises, especially interventions in emergencies (Government of Mauritius 2016). Nigeria is raising awareness on critical health issues that would enable community members to take pre-emptive actions against health challenges (Government of Nigeria 2014). The Nigerian government is working in close collaboration with other relevant government agencies, Mass Media and non-governmental organisations (NGOs). Climate-health education programmes are being promoted in schools to render good practices in community and personal healthcare under the changing climate. The government is expanding community health services delivery through establishing mobile clinics in dispersed settlements and difficult terrains (Government of Nigeria 2014).

Sierra Leone is providing community health education aimed at encouraging individuals to identify and eradicate breeding sites of vectors (Government of Sierra Leone 2016). Sierra Leone employs CHW and volunteers to educate the public in stress management, enhanced community education in the areas of food poisoning, hygiene and sanitation. Other health education programmes being carried out include the eradication of taxes on electric fans, food handling, rodent and pest eradication and community health inspections for mosquitoes. Similar health educational programmes are being considered by Botswana and Ghana through the Integrated Management of Childhood Infections (IMCI). This programme provides adequate public education on access to improved sanitation and safe water. Through this initiative, community health educators are employed as main health information providers at community level (Government of Botswana 2011; Government of Ghana 2015).

### Surveillance, research and monitoring

The Government of Gambia is designing health databases on diseases and various cases including those of communicable diseases (Government of Gambia 2012). User-friendly geographical information systems are being developed to provide real-time and remote data to assist with epidemiological description of systems in any place. Disease monitoring is being carried out to control infectious epidemic disease. Response and planning actions assist the government to provide public health interventions in a timely manner. The government is carrying out research in various locations to determine trends and patterns of diseases. Research has helped the government to investigate conditions that are favourable for breeding of vectors (mosquitoes) and spread of diseases (Government of Gambia 2012).

Egypt is embarking on research and disease surveillance through the gap identification approach. Surveillance and research provide satellite data essential to monitor disease patterns (Government of Egypt 2016). Research is being carried out on non-communicable diseases, heat-related sickness, water- and food-borne diseases and socio-economic impacts from extreme climatic events. Lesotho is considering developing monitoring and surveillance systems that include enhancing knowledge management to improve information sharing and communication networks (Government of Lesotho 2014). The government is establishing weather monitoring systems for adverse health-related conditions throughout the country. To reduce the vulnerability of the people from health hazards, the Government of Mauritius is considering preventative measures through strengthening the existing disease surveillance system (Government of Mauritius 2016).

In South Africa, through collaborative research, the government launched the Lubombo Spatial Development Initiative between Mozambique and Swaziland. The collaborative research led to a 70% decrease in malaria prevalence in Maputo Province (Mozambique) between 1999 and 2005 and a further 99% and 98% decrease in notified malaria cases in South Africa and Swaziland, respectively (Maharaj et al. 2016). The research aimed to intensify surveillance, community advocacy and training, control parasites using artemisinin-based combination therapy and vectors by indoor residual spraying using DDT or pyrethroids (Department of Environmental Affairs [DEA] 2011). Similarly, Seychelles is carrying out research to establish mosquito index distribution and associated impacts of the changing climate to communities. The work provides relevant data and information to predict the spread of diseases, epidemics and the impact of climate change (Government of Seychelles 2011). Eritrea has been working closely with ministries of transportation and communication, environment, energy and mines to promote research and surveillance for disease and vectors (Government of Eritrea 2012). Sierra Leone is considering promoting behavioural change and proliferation of rival habitats through entomological surveillance behaviours and epidemiological surveillance (Government of Sierra Leone 2016). The government is carrying out surveillance in communities vulnerable to diseases outbreaks for environmental sanitation purposes. Ghana and Botswana are strengthening their disease surveillance and response systems to improve health measures. This is being carried out through improved drainage, immunisation, sanitation and hygiene for vulnerable communities. Surveillance, research and monitoring is assisting the governments in the provision of ambulances for emergency health preparedness in vulnerable areas (Government of Botswana 2011; Government of Ghana 2015).

### Infectious, epidemic disease control programmes

Gambia is considering introducing vector control programmes through its planned health investment in education, social mobilisation and other prevention measures such as the use of insecticide-treated nets, mosquito repellents and low-cost anti-malarial drugs (Government of Gambia 2012). Vector control programmes will be implemented together with public awareness and health education programmes at community level. In addition, vector control programmes are incorporating climate science elements and climate change impacts on infectious and non-infectious diseases. The government is considering expanding vaccination programmes through its Expanded Programme of Immunisation to be administered to children from the age of 9 months throughout the country in all clinics. Similarly, Rwanda is implementing a nation-wide mosquito control programme to completely eradicate new infections from the falciparum parasite (Government of Rwanda 2012). The government is rolling out improved latrines and instituting a culture of handwashing with soap after toilet use in various institutions and households. As of 2012, 55% of households did not have access to improved latrines, with only 34% of households practicing handwashing after toilet use. Uganda formed the Epidemic and Disaster Preparedness and Response programme to spearhead infectious and epidemic disease control. The Malaria Control Programme is promoting effective and prompt malaria case management at household, health facilities and community levels. An example of direct programmes linking health to climate change interventions is the Highland Malaria Control Programme in Kabale District, which is linked to weather predictions and forecasting (Government of Uganda 2014).

Egypt is improving its vaccination programmes as part of its national epidemic disease control (Government of Egypt 2016). The programme is providing vaccinations for all school children and infants against diseases and is free of charge. Vaccination is also carried out for emerging diseases such as swine flu and human papilloma virus causing cancer cervix to vulnerable communities. Similarly, many other countries (e.g. Botswana, Eritrea and Sierra Leone) are promoting health programmes to control vector and epidemic disease (Government of Botswana 2011; Government of Eritrea 2012; Government of Sierra Leone 2016). In Africa, diarrhoea, cholera and typhoid are usually associated with water security issues (quantity and quality) (Government of Botswana 2011; Government of Egypt 2016; Government of Rwanda 2012).

### Policy development

Almost all countries sampled had some form of legal and policy provisions for sustainable health sector, although many were carried out in an era when climate change was not pronounced. Uganda developed health sector policies that include the Health Strategic Plan II (NHPII) (2005/2006–2009/2010) and the Health Policy of 2000 (Government of Uganda 2014). The government later updated the health policies to specifically address climate change impacts to the health sector. In this regard, the NHP II (as updated to the Health Strategic Plan (HSSP III) provides interventions and policy direction on climate change and human health. Furthermore, Uganda formed clusters on environmental health, disease prevention and health promotion to improve factors for environmental health. The HSSP III further mainstreams climate change matters through the formation of guidelines on climate change mainstreaming in the health sector, staff sensitisation on climate change and adaptation in the Ministry of Health (MoH) and other relevant departments, enhancing early warning systems and weather forecasts dissemination to health practitioners for timely response and preparedness (Government of Uganda 2014).

The South African government enacted its *Air Quality Act* (AQA) in 2007 in an effort to reduce greenhouse gas emissions and air pollutants. The AQA requires the development of air pollutants control plans at a national, provincial and local level as well as in urban centres. The country also developed the South African Air Quality Information System (SAAQIS) to provide all relevant information to climate change, legislation and air quality control initiatives. To meet the aim of the AQA, the government further developed the National Framework on Air Quality Management of 2007 to render medium- to long-term plan to meet the objectives of the AQA (DEA 2009).

Countries like Lesotho, Ghana and Namibia have developed National Adaptation Programmes of Actions (NAPAs) to address climate change, including impacts in the health sector (Government of Ghana 2015; Government of Lesotho 2014). Several programmes were developed addressing health concerns of climate change that included pests, vectors and disease control as well as water, sanitation and hygiene. The NAPAs outline disaster preparedness, epidemic and response to be spearheaded by the ministries of health and adopting WHO’s strategy on Integrated Disease Surveillance and Response. The governments have a multi-sectoral National Climate Change Policy to provide advisory role to relevant line ministries regarding policies on climate change issues and ministries responsible for environment.

The Government of Namibia passed its *Disaster Risk Management Act* (No. 10 of 2012) (Government of Namibia 2015). The main objective of the policy was to provide a framework on disease preparedness, prevention, response and recovery. The government established the Cholera Outbreak Response Team. The team comprises representatives from WHO, United Nations International Children’s Emergency Fund, the Namibian Red Cross Society and the Centre for Disease Control and Prevention. The team is controlling timely outbreaks of cholera and has established the Cholera Treatment Centres, provision of proper sanitation, safe water and health education (including food handling practices and personal hygiene) for the affected community (WHO 2014).

### Public health infrastructure and technology

The Government of Eritrea is working towards improving people’s accessibility to healthcare service. The MoH is cooperating and working with other ministries in ensuring adequate public health infrastructure to provide reliable transportation, safe and clean water, telecommunications and clean electricity (Government of Eritrea 2012). In addition, the MoH collaborates with the private sector engaged in health-related activities to provide best health services to its people. Similarly, Egypt is maintaining the national public health infrastructure. The country is working towards closing the gap in health indicators between its Lower Egypt and Upper regions and between rural and urban areas (Government of Egypt 2016). The lowest health indicators are found to be in the rural areas of Upper Egypt plagued with non-communicable and infectious diseases. From Egypt Demographic Health Survey (El-Zanaty & Way 2005), it emerged that the country is closing the gap by targeting 90% with access to improved health services. The country is working towards providing healthcare for managing coronary heart disease, complicated diabetes among rural areas and cancer (Government of Egypt 2016).

South Africa is promoting public health buildings through modifying architectural designs to suit weather changes and regimes (DEA 2011). The government is encouraging new building infrastructure that can curtail energy use and enable natural cooling. Roofs are being made stronger to be able to resist heavy storms and strong winds that can be disastrous to people. For example, at a 3.28 ºC rise in temperature, human health and comfort can be adversely affected (DEA 2011). In addition, The Gambia is promoting proper waste disposal to prevent toxic contamination and pathogenic during floods (Government of Gambia 2012). There are many technologies under consideration to reduce the impacts of the changing climate on human health populations. An example is in Kanifing Municipal Council (KMC), where there is improvement of public health infrastructure through the enforcement of building regulations and promotion of health-housing environment. Improved technological and preventative measures are being considered in high-risk areas where majority of people use untreated water. Sierra Leone is working on initiatives to change building designs to reduce heat stress (Government of Sierra Leone 2012). Other technological adaptation options include sustainable housing design standards in areas subjected to strong winds and high rainfall. A good example is that the government is encouraging people to paint their house roofs with white or silver to reduce heat absorption, cross-ventilation of windows and to pay attention to settlements design.

## Discussion

The results of this analysis provided a foundation that there are a number of adaptation measures being instituted by countries in sustaining public health systems under the changing climate. Many of the several activities being reported for this study as climate change adaptation were likely already underway as part of general health protection. In addition, some of the findings from the UNFCCC National Communications reports mirror traditional ways of dealing with the health systems in Africa. These interventions have continued to be used and scaled up with the understanding of adaptation in the era of climate change. Most African countries, especially those in lower resource settings, general health sector strengthening is a form of climate change adaptation. Similarly, in settings with high burdens of climate-sensitive health impacts, it can be difficult to distinguish climate change adaptation planning from general health sector planning. To this end, this can make adaptation planning difficult (Ford et al. 2014). Furthermore, several impacts that are likely to affect health systems are managed primarily by other sectors, and the health sector needs to collaborate closely with other sectors and ministries to effect comprehensive adaptation activities for health.

Many countries reported challenges faced while employing adaptation measures in the health sector. Financial constraints make the adoption of adaptation measures very slow. Although determinants such as financial resources are key for employing adaptation measures, other contextual factors are also significant, such as human perception. However, countries can access international funding for adaptation at the national level. This can be done, for instance, through the UNFCCC Adaptation Fund, Green Climate Fund and the Global Environmental Facility. Therefore, this analysis found that resource availability may impact countries’ engagement in adaptation processes. Policy and institutional factors are important as they affect how fully the adaptation process can be instituted (Kovats, Ebi & Menne 2003).

The study further revealed that African countries are also employing institutional structures focusing on environmental governance and other incentives that can enable them to be high adaptors to the changing climate. This is shown by countries such as Uganda, Lesotho, Ghana and Namibia that developed National Adaptation Programme of Actions as a way of addressing and implementing adaptation options including those for health sector. Although African countries lack financial resources, they are working towards health adaptation. The presence of obstacles that can hinder the capacity of adaption should be translated into health adaptation action.

The results further indicated that determining the progress of countries in employing adaptation measures with a large country sample is fruitful, including providing a good basis to formulate more complex hypothesis regarding institutionalising adaptation options by countries. Further analysis is crucial to understand the motive and drivers for adaptation and to understand the needs related to institutions and infrastructure for their implementation. The results have shown that low-income countries like those in Africa are willing to allocate their little finances and pay attention to adaptation issues affecting their development sectors such as health. These results were in line with reviewed literature suggesting that low-income nations are engaging in adaptation, and this can provide an obligation for developed countries to start facilitating transfer of technology and resources to developing nations.

The results also showed how African countries are participating in adaptation responses, international treaties and in health areas. This is explained by countries like Uganda, Ghana and South Africa that support other international treaties such as the World Health Assembly’s resolution (2008), which stipulates the need for member states to commit strongly on human health protection from climate change. Other treaties that are included in the Environmental Sustainability Index measure, of which African countries are signatories, are health-focused, addressing adaptation from environmental perspectives with a specific health component. For instance, International treaties such as the Montreal Protocol on Substances that Deplete the Ozone Layer has been regarded as successful in reducing negative environmental impacts (Mader et al. 2010). Our evidence suggested the need to include and establish the relationship between social spending, and countries’ priorities on sustainable adaptation measures, which in the near future must be an essential motive on developing model drivers for adaptation.

Decision makers are streamlining climate change impacts to highly planned adaptation emanating from people’s awareness on personal risks from the changing climate. The number of adaptation measures identified in this study depicts a strong relationship between people’s perceptions on the changing climate risks and adaptation. The established link could be attributed to reasons such as continued people’s views on climate change and its effects on their health status. This implies that there could be sufficient public perceptions and opinion across African countries in supporting climate change policy through the implementation of adaptation measures.

Other results showed that existence of extreme weather events stirs the willingness and motive for employing adaptation (Adger et al. 2013). The study provided a platform for future studies to systematically investigate the association between extreme weather events experience and adaptation. Moreover, the present study investigated only adaptation options employed by countries without investigating individual and private adaptation measures experiencing high and personal risk from the changing climate.

## Conclusion

This study reviewed adaptation measures contained in the UNFCCC’s National Communications from 21 African countries. The results indicate that there are a number of adaptation measures instituted by countries in promoting a sustainable public health sector, making it resilient to the changing climate. Key adaptation measures that emerged are weather-based forecasting and early warning systems, public education and awareness, surveillance, research and monitoring, enhanced infectious disease control programmes, policy development, improved public health infrastructure and technology.

There are various benefits reported from the implementation of such adaptation measures. These include increased international, regional and sub-regional cooperation; increased health benefits (improved latrines and the culture of handwashing after using the toilet, destroying breeding sites for mosquitoes); sustainable public health systems; improved water storage; increased public awareness on linkage between climate change, socio-economic and health, improved human health; institutional and human capacity development (through trainings of health professionals, etc.); strengthened friendly political, legislative and institutional framework in health mainstreaming into national planning improved health infrastructure and technologies. There is also increased geographical accessibility to health services; increased public education, surveillance, monitoring and reporting; increased access to safe water and improved sanitation; improved institutional organisation and capacity of researchers in all fields of climate change and health systems; as well as efficient monitoring and management of reported health cases. However, countries also reported challenges that can slow down the adoption of adaptation measures such as financial constraints, unfavourable policies, limited knowledge and slow diffusion of technology and innovation, poor attitude by communities in developing adaptation and development interventions.

Finally, the study shows that the rapid development of adaptation options as measures for managing the risks of climate change on human health has resulted in the emergence of a broad range of adaptation policies and management strategies in the African continent. Such adaptation options should be part of the bigger and ongoing climate change agenda in sustainable public health systems. However, the success of these initiatives is largely dependent on their acceptance and uptake by all involved stakeholders, which to date remains a significant challenge. Accordingly, policymakers require novel approaches on adaptation options to overcome barriers of sustainable health systems. The study noted that although there is no mention of adaptation to sea level rise and migration, either internal or trans-boundary, both are likely to be significant issues for certain regions of Africa as the climate changes.
